# Review of Cathode Plasma Electrolysis Treatment: Progress, Applications, and Advancements in Metal Coating Preparation

**DOI:** 10.3390/ma17163929

**Published:** 2024-08-07

**Authors:** Shuai Lu, Xiaowei Sun, Bowei Zhang, Junsheng Wu

**Affiliations:** Institute for Advanced Materials and Technology, University of Science and Technology Beijing, Beijing 100083, China; 15232507205@163.com (S.L.); xw_s920518@163.com (X.S.)

**Keywords:** cathodic plasma electrolytic treatment, metal coating, deposition parameters

## Abstract

Cathodic plasma electrolytic treatment (CPET) is an emerging surface modification and coating preparation technology. By utilizing plasma discharge induced through electrolysis and the cooling impact of electrolyte, metal cleaning, saturation, and coating preparation are efficiently achieved. In this review, the principle, application, and development of the CPET process are briefly summarized based on the past literature. Detailed insights are provided into the influence of electrolyte parameters (pH, metal salt concentration, and temperature), electrical parameters (voltage, duty cycle, and frequency), and process parameters (electrode area ratio, material, roughness, and deposition time) on plasma discharge and coating formation for metal coatings. The interaction mechanism between plasma and material surfaces is also investigated. Recommendations and future research avenues are suggested to propel CPET and its practical implementations. This review is expected to provide assistance and inspiration for researchers engaged in CPET.

## 1. Introduction

Surface coatings have essential applications in materials science and engineering, such as enhancing the material’s corrosion and wear resistance and imparting other functional characteristics, making it better meet various usage requirements [1,2]. In industry, some of the more mature coating preparation techniques include physical vapor deposition (PVD) [3], chemical vapor deposition (CVD) [4], spray coating technology [5], electroplating [6], and hot-dip galvanizing [7]. Although the above techniques have been extensively utilized, they suffer from complex pre-treatment processes, low deposition rates, and environmental issues. Therefore, there is a growing urgency to develop new coating preparation technologies to minimize environmental impacts and enhance production efficiency.

Plasma electrolysis treatment (PET) has emerged in the last decades as an alternative to metal surface treatment [8,9]. The PET process produces four effects at the same time: strong electric field, oxidizing free radicals, ultraviolet light, and shock wave, which has attracted increasing attention in the fields of material cleaning, etching, film deposition, and heat treatment [10,11,12]. Currently, two main types of PET processing have been developed: anodic plasma electrolytic technology and cathode plasma electrolysis technology. Anodic plasma electrolytic technology, known as plasma electrolytic oxidation (PEO), has become a mature surface treatment technique after decades of in-depth research [13,14,15]. In contrast, the cathode process is still far behind in fundamental understanding, process parameter control, and surface analysis.

Cathodic plasma electrolytic treatment is an emerging surface technique based on liquid-phase plasma discharge [16,17,18]. The liquid medium is electrolyzed by applying the potential between the cathode workpiece and the counter electrode to produce high-energy plasma (micro-arc discharge on the workpiece surface) [19]. In parallel with the plasma discharge, bubbles surrounding the cathode workpiece surface periodically form and collapse, generating powerful shock waves [20]. Under the combined physicochemical effects of plasma acceleration, ion adsorption, and bubble rupture, the substrate’s cleaning, deposition, or surface modification is recognized. Compared with the traditional electrochemical treatment technology, CPET eliminates the boundary layer diffusion and possesses the natural advantages of the eco-friendliness, high efficiency, and easy preparation of nanocrystalline structures.

In the past decade, CPET has been successfully used to apply various metal coatings (such as Zn [21], Ni [22], Mo [23], Cr [24], Co [25], etc.) and ceramic coatings (such as Al_2_O_3_ [26], ZrO_2_ [27], etc.). It has been found that the CPET process is influenced by many parameters, such as electrolyte parameters, processing time, temperature, power supply parameters, etc. By reviewing the literature on CPET, the present paper summarizes its development of CPET and briefly describes its principles and applications. In particular, some specific examples elaborate on the effects of the electrolyte, power supply, and process parameters on the preparation of metal coatings by CPET. In the end, the current understanding of the deposition mechanism and plasma effects of CPET is presented.

## 2. Principles of CPET

**Figure 1:**
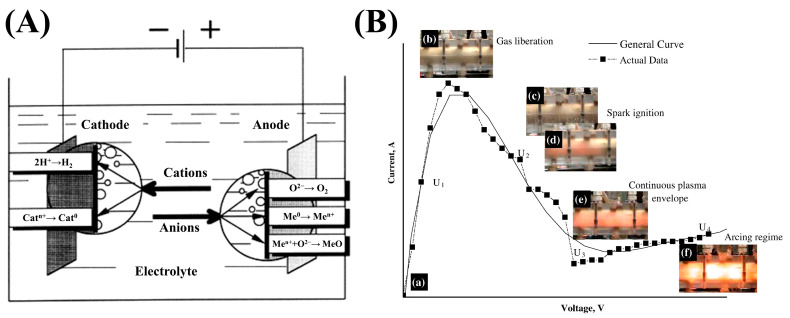
(**A**) Schematic diagram of the CPET process [8]; (**B**) Typical current–voltage curves for CPET [9]: (a) Current−voltage curve during CPET process; (b)–(f) Photos of bubbles and plasma with increasing voltage.

As shown in Figure 1A, a typical CPET process consists of two electrodes, where the workpiece serves as the cathode and the auxiliary electrode serves as the anode (usually an inert electrode) [8]. Both electrodes are immersed in an electrolyte (e.g., NiSO_4_ or ZnSO_4_ aqueous solution) and are spaced a few centimeters apart. By applying a pulsed or continuous direct current (DC) power supply, plasma electrolysis is initiated when the potential difference between the electrodes reaches a critical level. The typical current–voltage characteristic curves in the CPET process are shown in Figure 1B [9].

In the U_1_ region, the current increases linearly according to Faraday’s law. At this stage, a hydrogen evolution reaction occurs, while the electrolyte is rapidly vaporized by Joule heat and mixed with hydrogen. As the voltage increases, the rate of bubble formation on the cathode surface gradually increases, leading to the formation of an unstable gas film [9]. The formation of the unstable gas film reduces the contact area between the electrolyte and the cathode, resulting in the limiting current value. From U_2_ to U_3_, the thickness and stability of the gas film increases as the current continues to decrease. In the subsequent U_3_ region, a relatively stable gas film is fully formed, and a stable plasma discharge is observed. Therefore, the U_3_–U_4_ region is generally used as the working region for CPET. Kellogg first proposed this empirical method of deposition voltage selection. Hence, the U_3_–U_4_ region is also known as the Kellogg zone [28]. In the Kellogg region, the near-surface of the substrate (coating) is heated to a molten state due to plasma action, while the surrounding lower-temperature electrolyte quenches the substrate (coating). The periodic melt–quench interaction results in a unique microstructure and excellent bonding of the CPET-prepared coatings.

## 3. Development of CPET

In previous research, cathodic discharge was considered a byproduct of anodic plasma discharge processes and was not systematically studied. With the establishment of liquid-phase discharge theories and the success of anodic plasma electrolysis technology, CPET has gradually gained attention from researchers.

The discovery of the cathode plasma discharge phenomenon can be traced back to 1950. Kellogg et al. electrolyzed a sulfuric acid solution using platinum wire at high current densities. When the applied voltage exceeded a critical value, the current–voltage relationship deviated from the normal Faraday’s law, and the surface of the cathode emitted a bright blue light. Since this discharge phenomenon is very similar to the “anodic effect”, it was named the “aqueous cathodic effect” [29]. In 1964, A. Hickling et al. analyzed in detail the glow discharge electrolysis process. Cathodic discharge was considered a form of electrolysis involving energy transfer and charge transfer. The involvement of plasma helps break down solvent molecules into reactive radicals, facilitating electrochemical reactions [30]. At the same stage, some basic theoretical and practical research on cathode plasma discharge was carried out simultaneously [31,32]. All studies have found a common denominator: a significant deviation from the normal Faraday electrolytic state occurs when the potential reaches a critical value, inducing a cathode plasma discharge. The impressed potential, temperature, electrolyte composition, hydrodynamics, electrode material, and geometry influence the formation and evolution of the continuous plasma around the electrode.

As the potential value of liquid-phase plasma discharge technology is gradually recognized, a large number of Russian researchers were engaged in basic and theoretical research in related fields. In 1997, Steblianko and Ryabkov introduced the cathodic plasma discharge effect into the field of surface engineering and developed cleaning and deposition processes [33]. In 1999, Yerokhin systematically summarized the significant results published by predecessors and reviewed in detail the fundamental theories of plasma electrolysis in surface engineering [8]. In addition, the term “plasma electrolysis technology” was used to name this technology. At the same time, subdivision processes such as plasma electrolytic oxidation (PEO) and plasma electrolytic saturation (PES) were introduced, which laid a foundation for the application and development of plasma electrolysis technology.

**Figure 2:**
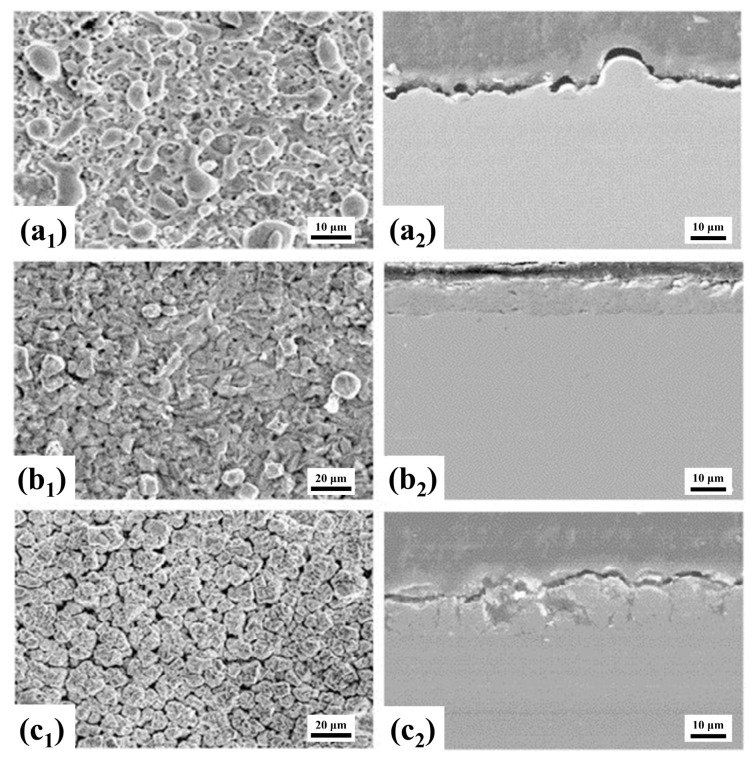
(**a_1_**,**a_2_**) Cleaning; (**b_1_**,**b_2_**) Zn coating; (**c_1_**,**c_2_**) Zn-Al coating [34].

In 2002, Meletis et al. carried out related work on the cleaning of metal surface oxide films and the preparation of metal coatings [34]. Figure 2(a_1_–c_2_) shows the clean surface, Zn coating, and Zn-Al coating prepared by Meletis et al., respectively. As shown in Figure 2(a_1_,a_2_), the clean metal surface prepared by CPET has unique anchor profiles, which provide an ideal surface for subsequent coating deposition. In addition, Figure 2(b_1_–c_2_) shows that the CPET process allows for the production of dense and uniform metal and alloy coatings, demonstrating enormous potential as a novel plasma surface engineering technology. In 2005, based on the current–voltage curve during deposition, Gupta et al. proposed a process mechanism for preparing metal coatings via CPET [9]. This mechanism effectively elucidates the high deposition rate of CPET, aligning well with the characteristic points of the current–voltage curve, and has gained widespread acceptance. With the continuous improvement of CPET-related theories, research on the parameters of metal coatings, ceramic coatings [35,36,37,38], and cleaning [39,40,41] has emerged. These parameters include treatment voltage (duty cycle, frequency), treatment time, electrode material, electrolyte (composition, concentration, and pH), etc. The main outcome of this stage is the preparation of a series of coatings, such as C [42], ZrO_2_ [43], ZrO_2_-Y_2_O_3_ [44,45], Al_2_O_3_-Y_2_O_3_ [46], Ni-P [47], etc. At the same time, some researchers have explored the principles and mechanisms of CPET through experiments and theoretical simulations to deepen the understanding of the deposition process [48,49].

**Figure 3:**
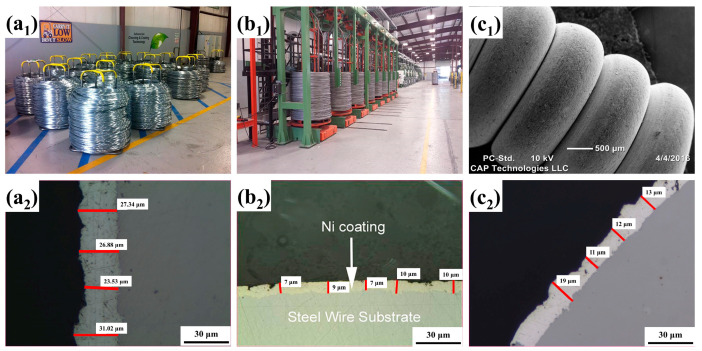
(**a_1_**,**a_2_**) Zn coating; (**b_1_**,**b_2_**) Ni coating; (**c_1_**,**c_2_**) Ag coating [50].

In 2011, the CAP company (Los Angeles, CA, USA) successfully pioneered commercialization by combining CPET with continuous preparation of coatings. The CAP company uses a multi-chamber device, supplemented by a prefabricated foam electrolyte, to determine the continuous preparation of a variety of metal coatings on the wire. Figure 3(a_1_–c_2_) are the Zn, Ni, and Ag coatings fabricated by CAP, respectively [50]. Under high-temperature plasma and cyclic quenching, the coatings are tightly bonded to the substrate with uniform thickness and good densification, showing excellent coating quality. With the success of commercialization, CPET is gradually developing towards intelligent manufacturing. Establishing a connection between plasma physics, materials science, and computer science using static and dynamic neural network methods is beneficial for promoting the development of intelligent electrolytic plasma technology.

## 4. Evolution of CPET Devices

**Figure 4:**
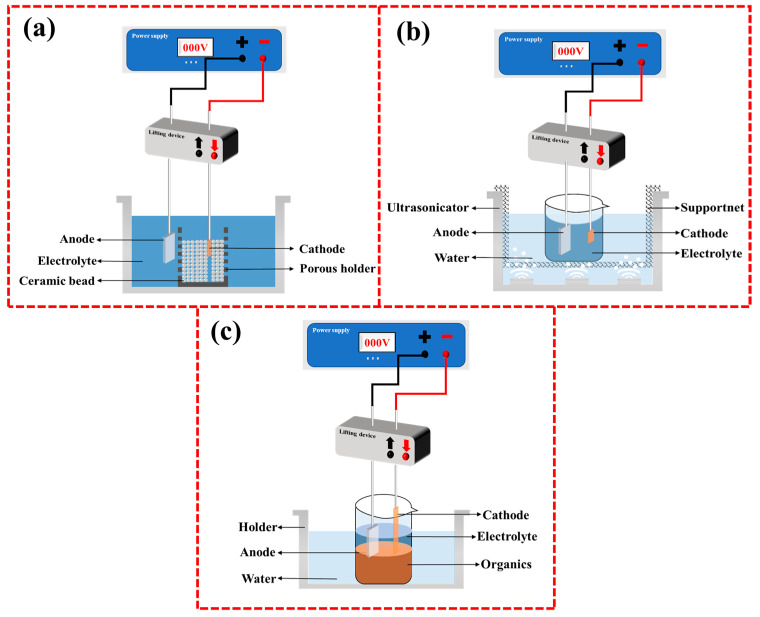
Variants of traditional immersion devices: (**a**) Microbead-assisted device; (**b**) Ultrasound-assisted device; (**c**) Layered solution device.

With the continuous advancement of CPET technology, CPET devices are also constantly being improved. The mainstream deposition equipment mainly includes the traditional immersion type and porous droplet type. The traditional immersion type has strong compatibility with the shape of the substrate, making it suitable for substrates of complex shapes. However, it faces issues of high process energy consumption and unstable plasma arcs, so it is more suitable for smaller samples. To reduce energy consumption and improve process stability, many variants have been developed based on traditional immersion devices, such as microbead-assisted devices [51] and ultrasound-assisted devices [52]. Deng et al. studied the impact of adding microbeads to the traditional immersion device, with the experimental setup shown in Figure 4a. The results show that introducing microbeads near the cathode significantly reduces the cathode current density required for plasma discharge [51]. Adding microbeads effectively suppresses the transport of electrolyte bubbles near the cathode, making the electric field distribution around the cathode more uniform, thus inducing the formation of a thin and uniform gas film. As a result, stable and gentle plasma discharge occurs across the entire cathode surface rather than an intense plasma discharge, effectively constraining the plasma discharge process. One study by our research group applied ultrasound assistance based on a traditional immersion device, with the experimental setup shown in Figure 4b [52]. After applying ultrasound, bubbles in the gas film are mechanically broken down into microbubbles, promoting a constant high-speed replacement of bubbles in the gas film. The reduction in bubble size and the high-speed iteration allow for a more uniform distribution of the gas film on the cathode surface, inducing uniform and random plasma discharge. Additionally, Zhang et al. designed a layered solution device to prepare large-area coatings, as shown in Figure 4c [22]. The solution is divided into a transparent electrolyte upper layer and a high-density CCl_4_ organic solvent lower layer. This layered electrolyte strategy effectively ensures the discharge area of the electrode, providing new insights for the continuous preparation of large-area coatings by CPET.

**Figure 5:**
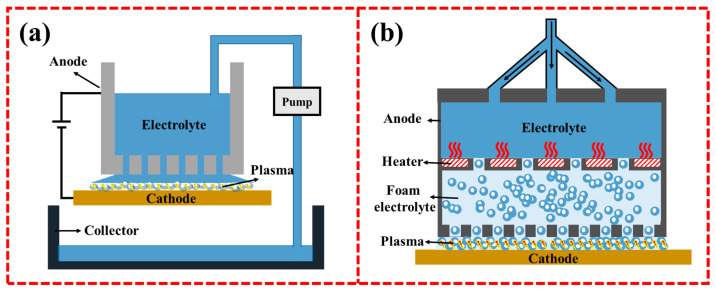
(**a**) Dripping type device; (**b**) Multi-chamber device by the CAP company.

The dripping type device is usually paired with mobile equipment, allowing for the treatment of large sheet steel and wire (Figure 5a). The CAP technology company has improved this device over several generations of technical iterations and designed a mature multi-chamber deposition system, which achieves continuous cleaning of wires and coating deposition [53,54]. As shown in Figure 5b, the multi-chamber device consists of one or more anode heating chambers separated by porous plates. The electrolyte is heated to its boiling point through multiple heating chambers and finally enters the working gap as a foam electrolyte. By using this multi-chamber design, the traditional electrolyte is transformed into a foam electrolyte, which reduces the current during the plasma discharge process, thereby reducing power consumption and enhancing economic efficiency. Furthermore, the foam content can be adjusted by controlling the heating process of the electrolyte, achieving the controllable regulation of the plasma discharge process.

## 5. Application Progress of CPET

**Figure 6:**
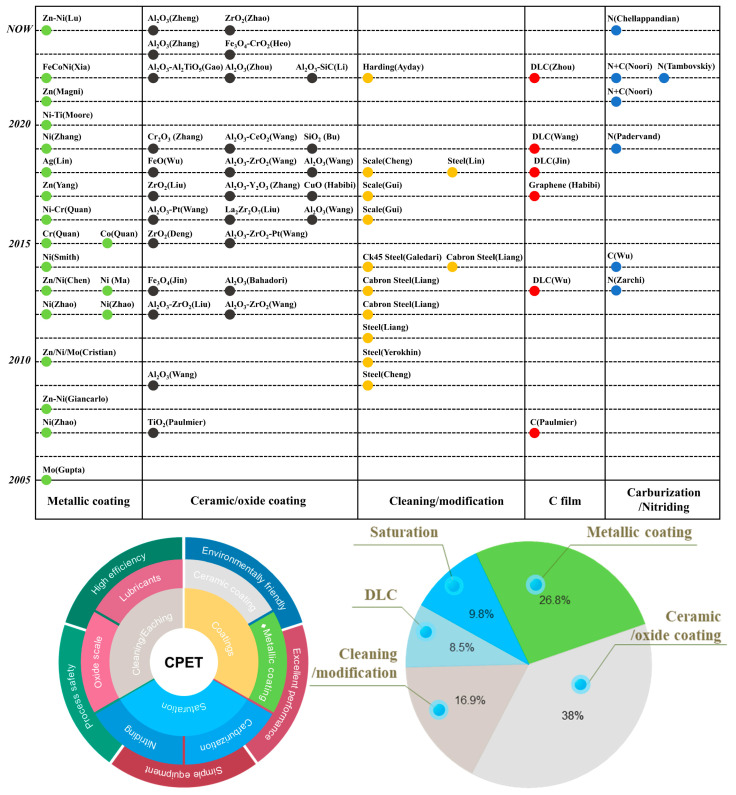
Summary of the application of CPET.

During the CPET process, strong electric fields, oxidative free radicals, ultraviolet light, and shock waves are all generated simultaneously, which is the fundamental reason for its high application value. Figure 6 summarizes the progress of applications using CPET for coating deposition, cleaning, and surface modification since 2005. Most applications focus on coating preparation, such as metal (alloy) coatings, ceramic (oxide) coatings, and diamond-like thin films. Additionally, relatively extensive research has been conducted on surface oxide film cleaning, carburizing, and nitriding applications.

(1) Surface cleaning

Metal surface cleaning is essential in modern manufacturing processes. Oxide films, dirt, oil, or chemicals on metal surfaces can seriously affect the effectiveness of subsequent surface treatments. At present, conventional cleaning methods include sandblasting [55,56], water jet [57,58], ultrasonic cleaning [59,60], acid/lye cleaning, emulsion cleaning, etc. However, these methods require multiple cleaning steps and produce harmful particles or chemicals. In contrast, surface cleaning with CPET has the advantage of being cost-effective and environmentally friendly [61].

**Figure 7:**
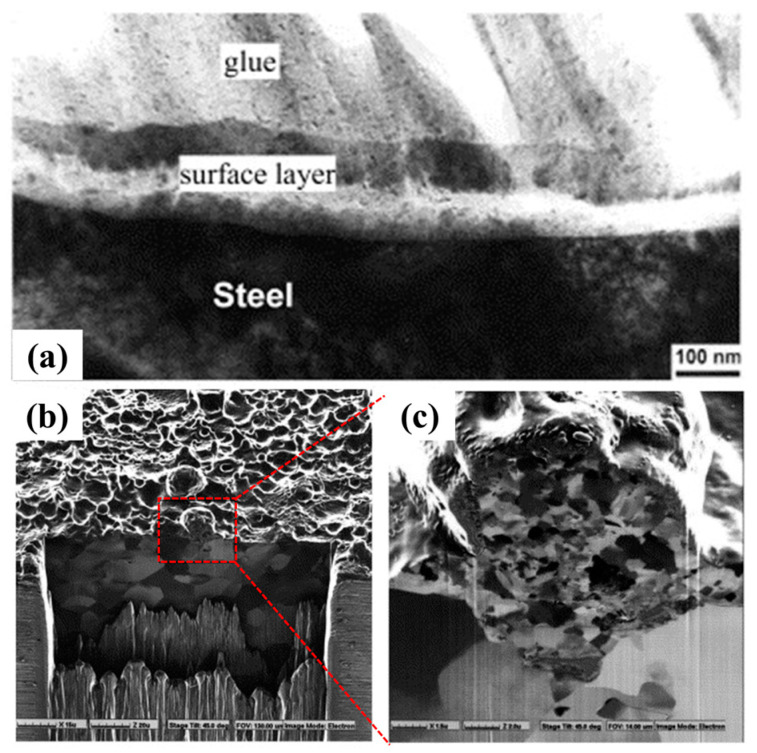
(**a**) Cross-sectional morphology after cleaning; (**b**,**c**) the ultra-fine structure after cleaning [34].

Figure 7 shows the cross-sectional TEM morphology of AISI 1010 low carbon steel after cleaning in a 14 wt.% NaHCO_3_ aqueous solution [34]. As shown in Figure 7a, impurities and rust on the metal surface can be directly removed by the CPET cleaning strategy. During the cleaning process of CPET, the metal surface melts to a certain extent under the action of high-temperature plasma, and the hydrogen bubbles produced by electrolysis are broken down by plasma. The shock wave generated by bubble rupture is beneficial in removing the oxides on the metal surface, thus forming a clean surface. At the same time, through the discharge of high-energy plasma on the material’s surface and the cyclic quenching of electrolytes, a thin layer of nanocrystals can be formed while removing the surface impurities (Figure 7b,c), significantly improving the material’s corrosion resistance.

(2) Surface modification

Traditional surface modification techniques such as ion implantation physical or chemical vapor deposition are generally carried out under vacuum conditions. Plasma electrolytic saturation (PES) is an attractive and flexible process that can prepare the saturated layer under atmospheric pressure. Compared with the traditional surface modification process, PES has the advantages of short treatment time, small environmental impact, and low-temperature operation. At present, a variety of saturated layers have been prepared by the PES process, such as plasma electrolytic carburizing (PEC), plasma electrolytic nitriding (PEN), plasma electrolytic nitriding (PEB), plasma electrolytic carburizing (PEC/N), etc. [62,63,64]. Under the action of high-energy plasma bombardment, PES can obtain a saturated layer on the metal surface with high efficiency. In addition, the excessive growth of grains in the saturated layer is limited by the cyclic effect of plasma heating and quenching cooling. According to the published work, the saturated layer prepared by PES generally has a nanocrystalline structure with excellent wear and corrosion resistance. PES is simple to operate and has no additional restrictions on the material and shape of the matrix, showing good advantages in actual production [65,66,67,68].

(3) Ceramic (oxide) coating

**Figure 8:**
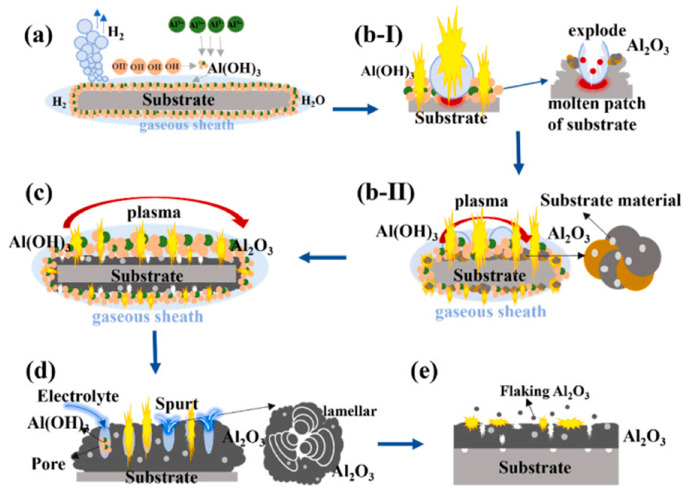
Schematic diagram of the deposition process of the Al_2_O_3_ coating [18]: (**a**) and (**b-I**) The formation of Al(OH)_3_ colloidal particles; (**b-II**) Al(OH)_3_ dehydrates to form Al_2_O_3_; (**c**) Al_2_O_3_ melts to form a coating; (**d**) Injection, sintering and solidification of Al_2_O_3_; (**e**) Spark fading stage.

Ceramic coatings are widely used as thermal barrier coatings for Ni and Ti alloys due to their high temperature and pressure resistance [69,70,71]. Currently, PEO technology based on anodic plasma discharge effect is the primary method of preparing ceramic coatings. However, the coating prepared by PEO is usually limited to depositing on valve metals such as Ti and Mg, and its composition is mainly composed of oxides of the matrix [72,73]. In contrast, CPET is suitable for most conductive substrates, and the composition of the deposits can be regulated by designing electrolytes. Due to the advantages of low cost, high efficiency, adjustable microstructure, and vital inclusiveness, a series of ceramic (oxide) coatings, such as Al_2_O_3_ and ZrO_2_, were prepared by CPET. Among them, the Al_2_O_3_ coating is the most widely studied. Figure 8 shows the schematic diagram of the deposition process of the Al_2_O_3_ coating prepared by CPET. The deposition of the Al_2_O_3_ coating undergoes a combination of processes, including plasma discharge, molten Al_2_O_3_ spray, sintering, solidification, and stacking [18]. The process of spray, sintering, and solidification promotes the thickening of the Al_2_O_3_ coating. When the coating reaches a certain thickness, the plasma discharge cannot completely break down the Al_2_O_3_ coating, and the plasma discharge intensity decreases. At this time, the CPET process enters the stage of spark extinction. Generally, the Al_2_O_3_ coating prepared by the CPET method is composed of a mixed crystalline phase of α-Al_2_O_3_ and γ-Al_2_O_3_, and the surface exhibits a typical porous and rough microstructure [16,74,75]. Therefore, improving the porosity and surface roughness of the ceramic coating prepared by CPET is the key to further enhancing its performance.

(4) Metal (alloy) coating

Currently, the preparation of metal coatings mainly adopts the electrodeposition method. In the traditional Faraday electrolysis zone, a good quality metal coating can be prepared by electrodeposition at a small voltage. Although electrodeposition has the advantages of simple equipment and a mature process, its pre-treatment process is complicated and requires steps such as grinding, polishing, degreasing, and acid activation of the sample. In contrast, CPET does not require additional pre-processing steps. For a sample with a poor surface state, the preparation of the metal coating can be quickly achieved by combining plasma cleaning and deposition. In addition, the electrolyte composition of the CPET process is relatively simple, and high-speed deposition of metal coatings can be achieved without adding surfactants and other organic chemicals.

**Figure 9:**
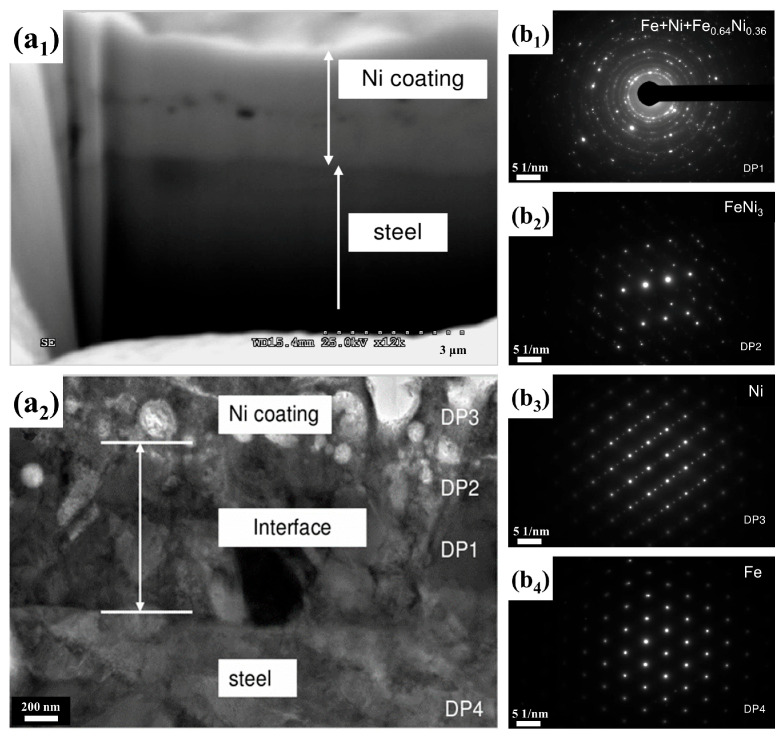
Cross-section TEM characterization of a Ni coating prepared by CPET [28]: (**a_1_**) Low magnification morphology of Ni coating; (**a_2_**) High magnification morphology of Ni coating; (**b_1_**–**b_4_**) are the diffraction patterns of the (DP1)–(DP4) regions, respectively.

A variety of metal coatings have been prepared by CPET, including monometallic coatings (such as Ni [22], Zn [34], Co [25], Cr [24], Mo [23], Cu, etc.) and binary coatings (such as Ni-Cr [76], Zn-Ni [77], Zn-Al [34], etc.). The preparation of metal coatings by CPET has the advantages of a high deposition rate, nanocrystalline structure, and excellent adhesion with the substrate. The deposition rate of Zn coatings prepared on medium-carbon steel using the CPET method can reach 1 μm/s [9]. In addition, due to the high temperature of plasma, a good diffusion bond layer can be formed between the metal coating and the substrate, which has a good adhesion. Figure 9 illustrates the cross-sectional TEM characterization of Ni coatings prepared by CPET. A Ni coating prepared by CPET tends to have two sublayers: the top layer consists of almost 100% coating material, and the thin transition layer below consists of a mixed phase of deposited metal and matrix. The surface of the metal coating deposited by the plasma method has an ultra-fine nanocrystalline structure, which imparts excellent corrosion resistance and mechanical properties to the coating [28].

## 6. Research Progress on the Preparation of Metal Coatings by CPET

With the continuous progress of science and technology, metal materials are increasingly and widely used in electronics, automotive, and aerospace fields, and the requirements for the hardness, wear, and corrosion resistance of metal materials are also increasing [78]. The CPET process can rapidly prepare various metal or alloy coatings with nanocrystalline structure and excellent adhesion, which can effectively improve the stability of the substrate in harsh environments and broaden the application scenarios of materials. Currently, the research on the preparation of metal coatings by CPET mainly focuses on the effects of power electrolyte composition, supply parameters, and process conditions on the surface morphology, microstructure, and properties of the coatings. At the same time, the mechanism of CPET preparation of metal coatings has also been studied. 

**Figure 10:**
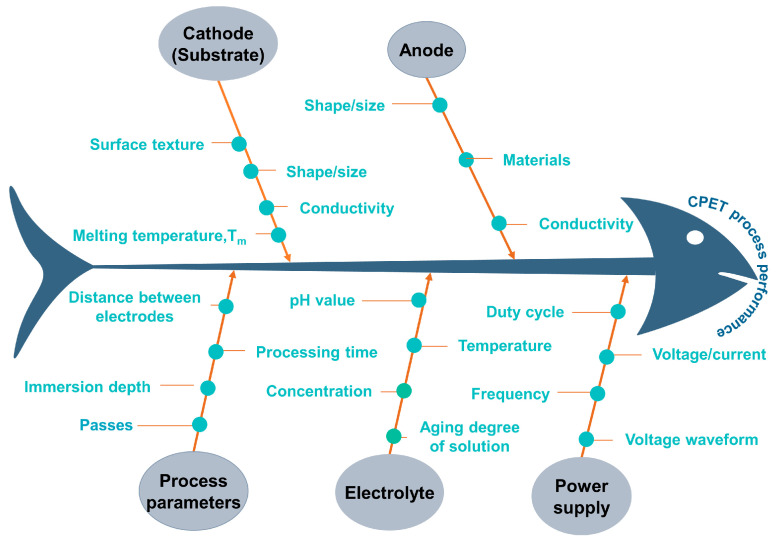
Influencing factors of metal coating preparation by CPET.

The CPET process is a complex electrochemical process involving multiple physical fields containing a large number of process parameters that simultaneously affect process performance. Figure 10 shows almost all parameters that may affect the CPET process in the form of a fishbone diagram to illustrate causal relationships. Electrolyte parameters include electrolyte type, metal salt concentration, temperature, etc. Electrical parameters include deposition voltage, duty cycle, and frequency, etc. In addition, the quality of metal coatings prepared by CPET is also affected by parameters such as electrode material, electrode surface roughness, and deposition time. The following is a comprehensive review of the above parameters for CPET’s preparation of metal coatings.

### 6.1. Electrolyte Parameters

(1) Type of electrolyte

**Figure 11:**
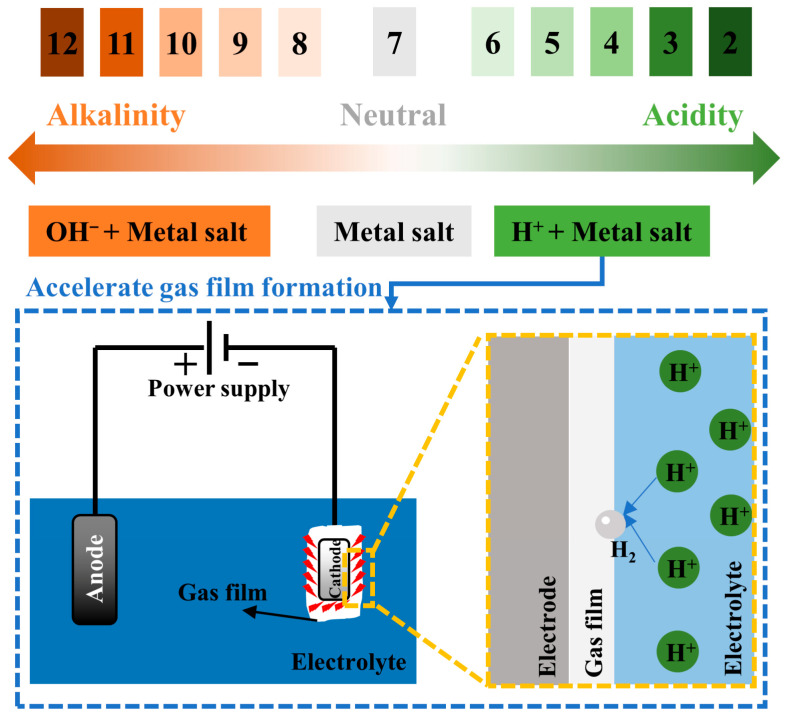
Electrolyte categories in the preparation of metal coatings by CPET.

The CPET process involves gas evolution, metal deposition, and other competing reactions. Compared with the solid–liquid two-phase interface of traditional electrodeposition, the CPET process involves a more complex solid–liquid–gas three-phase interface. As the “precursor” of the gas film, the characteristics of the electrolyte directly affect the uniformity and stability of the gas film [79,80]. As shown in Figure 11, the types of electrolytes used to prepare metal coatings by CPET can be divided into three categories according to pH: acidic, neutral, and alkaline.

Ma et al. deposited Zn coatings in water and water–ethanol (C_2_H_5_OH) neutral electrolyte systems [81]. The stable plasma voltage of a water–ethanol mixed solvent system is significantly lower than that of a pure water solvent system, and the initial discharge voltage decreases with the increase in ethanol content. Although adding ethanol is beneficial to reducing deposition voltage, it does not effectively improve the shortcomings of coarse particles and the compactness of Zn coating in a neutral electrolyte system. Yang et al. prepared Zn coatings using a neutral electrolyte system containing only ZnSO_4_ [82]. The thickness and density of the zinc coating can be adjusted to a certain extent by changing the electrical parameters. However, the final zinc coating is still rough and porous, with defects and holes on the surface. Therefore, the preparation of a metal coating by CPET in a neutral electrolyte system can be achieved, but the high deposition voltage and unstable plasma discharge lead to poor coating quality. Currently, acidic metal salt solutions are often used as electrolytes to improve the deposition process and coating quality.

**Figure 12:**
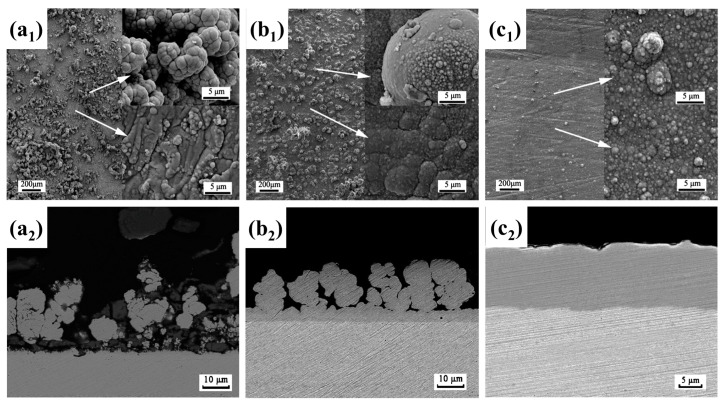
Effect of H_2_SO_4_ concentration on surface and cross-sectional morphology of Ni coating: (**a_1_**,**a_2_**) 0 g/L; (**b_1_**,**b_2_**) 20 g/L; (**c_1_**,**c_2_**) 40 g/L [83].

Zhao and colleagues explored the impact of the H_2_SO_4_ concentration on the surface and cross-section morphology of Ni coatings produced via CPET [83]. The large amount of H^+^ introduced by adding H_2_SO_4_ could accelerate gas film formation and plasma discharge formation. Adding H_2_SO_4_ affects the discharge order during the deposition process, affecting the deposition rate. Under a low hydrogen ion concentration, the current is mainly conducted through Ni^2+^ and SO_4_^2−^ in the electrolyte, and Ni^2+^rapidly deposits on the cathode surface, resulting in a loose and uneven coating structure (Figure 12(a_1_–b_2_)). As the concentration of H^+^ increased, the discharge of H^+^ on the cathode surface led to a significant decrease in the deposition efficiency of Ni, which, in turn, improved the coating morphology (Figure 12(c_1_,c_2_)). This result aligns well with Quan et al.’s work on Co coating preparation [25]. Adding H_2_SO_4_ reduces the current density and deposition rate, contributing to the formation of a uniform and dense Co coating.

Compared with neutral and acidic electrolytes, alkaline electrolytes in preparing metal coatings by CPET are still in their infancy. For some metal salts (such as AgNO_3_), the stability of neutral and acidic electrolytes is poor. Therefore, it is more suitable for coating deposition in alkaline electrolytes. Lin et al. prepared nano-silver coatings by CPET in an alkaline ammonia system [84]. The main components of the electrolyte are deionized water, KNO_3_, AgNO_3_, and ammonia. When deposited in a neutral or acidic electrolyte environment, the cathode surface is prone to generate gray-brown silver oxide intermediates, which hinders the deposition of silver ions, resulting in the inability to obtain a uniformly distributed silver coating. In the ammonia system, intermediates such as silver oxide interact with ammonia to form a diammonium hydroxide silver complex, which enhances the stability of the electrolyte and accelerates the plasma electrolysis reaction rate.

(2) Metal salt concentration

**Figure 13:**
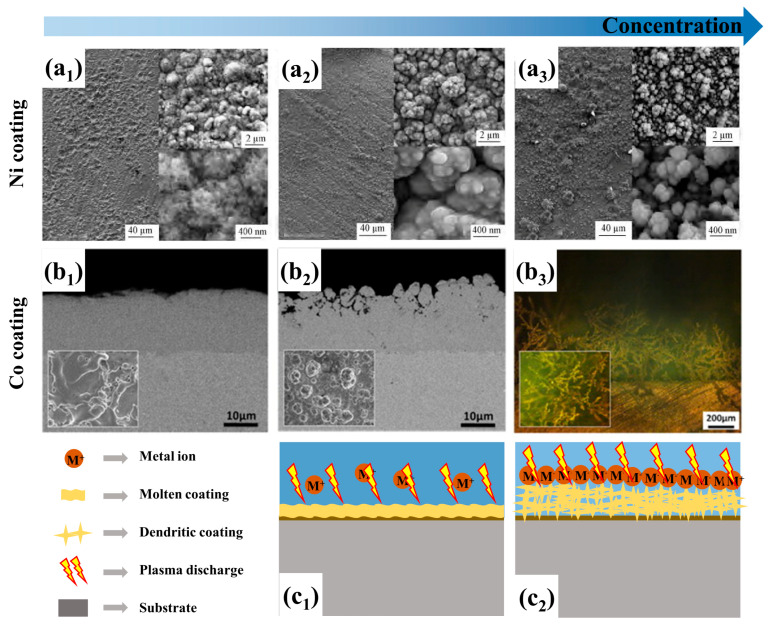
Effect of metal ions concentration on surface and cross-section morphology: (**a_1_**) 50 g/L; (**a_2_**) 100 g/L; (**a_3_**) 150 g/L NiSO_4_ [85]; (**b_1_**) 10 g/L; (**b_2_**) 80 g/L; (**b_3_**) 250 g/L CoSO_4_ [25]; (**c_1_**,**c_2_**) Schematic diagram of low and high concentration deposition, respectively.

In the CPET process for preparing metal coatings, the concentration of metal ions in the electrolyte directly influences the coating’s deposition rate and surface melting degree. The interplay between deposition rate and melting degree collectively shapes the coating’s topography, density, and properties. Figure 13(a_1_–b_3_) displays the Ni coating and Co coating prepared at varying concentrations of metal salts, respectively. As shown in Figure 13(a_1_,b_1_), the Ni [85] and Co coatings [25] are dense under low metal salt concentration conditions, and the melting morphology resulting from high-temperature plasma is clearly visible on the coating’s surface. Figure 13(a_2_,b_2_) illustrates that the coating structure gradually becomes looser with an increase in metal salt concentration, and the porosity increases. When the concentration of metal ions is excessive, the coating is mainly composed of loose dendritic structures, presenting a three-dimensional porous morphology (Figure 13(a_3_,b_3_)). In fact, the deposition rate of coating is significantly influenced by the concentration of metal salts. At low metal salt concentrations, the rate-controlling step of the deposition process is the mass transfer of metal ions in the electrolyte. The deposited material formed on the substrate can be fully melted by the plasma to create a dense and uniform coating structure (Figure 13(c_1_)). When the concentration of metal salts exceeds a specific limit, a large number of metal ions in the electrolyte act as carriers for the transfer of current, leading to rapid deposition and loose crystallization (Figure 13(c_2_)). According to the indexes of surface porosity and section thickness, the electrolyte components and supporting process parameters of various metal coatings with better quality are summarized in Table 1.

(3) Electrolyte temperature

The electrolyte temperature significantly impacts the arc initiation voltage, deposition rate, surface topography, and other aspects. Generally, preheating the electrolyte is favorable for forming a plasma arc on the cathode surface. West and Jarhav observed an increase in electrolyte conductivity from 345 mS cm^−1^ to 650 mS cm^−1^ as the electrolyte temperature increased from 35 °C to 80 °C [90]. The rise of electrolyte conductivity induces more hydrogen bubbles to form near the cathode. The increase in the number of hydrogen bubbles reduces the arc voltage, increasing the rate and intensity of plasma generation. In addition, the increased ion transport rate at high temperatures makes it easier for metal ions to be reduced to metal at the cathode. Therefore, the deposited film’s surface morphology can be controlled by controlling the electrolyte’s temperature. It should be noted that a too high temperature may lead to volatilization and loss of electrolytes and affect electrolysis.

### 6.2. Electrical Parameter

(1) Deposition voltage

**Figure 14:**
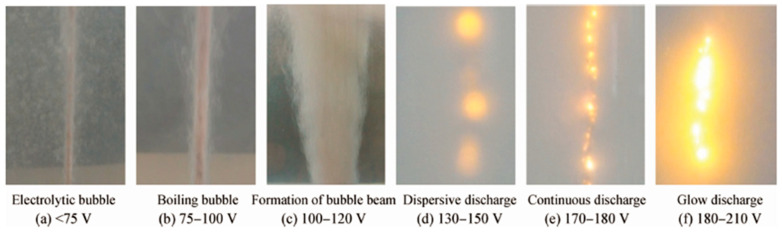
Cathode surface state under different voltages [81].

Operational voltage is crucial for plasma discharge generation and coating deposition efficiency. In the CPET process, increasing the voltage beyond a critical level is essential for stable coating deposition. Various factors affect the critical voltage, such as the electrodes’ conductivity, the electrolyte’s concentration, the distance between the electrodes, etc. Within a specific range, an appropriately high voltage will induce a more dense and intense plasma discharge, thereby increasing the deposition efficiency of the metal coating. As depicted in Figure 14, plasma arc intensity and density intensify with increasing voltage [81]. Nevertheless, exceeding a certain voltage threshold can significantly compromise coating surface quality, leading to micropore formation.

**Figure 15:**
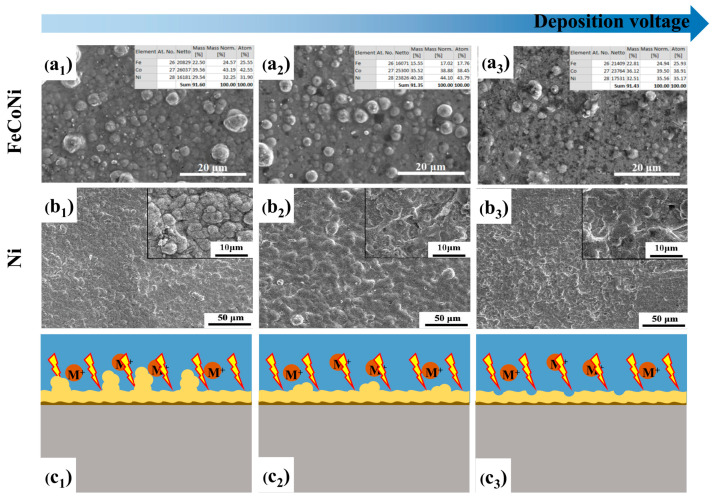
Effect of deposition voltage on surface topography: (**a_1_**) FeCoNi-100V; (**a_2_**) FeCoNi-110V; (**a_3_**) FeCoNi-120V [91]; (**b_1_**) Ni-110V; (**b_2_**) Ni-125V; (**b_3_**) Ni-155V [85]; (**c_1_**–**c_3_**) Schematic diagram of low, suitable and excessive deposition voltage, respectively.

Zhuang et al. investigated the effect of deposition voltage on preparing ternary FeCoNi coatings by CPET [91]. As the deposition voltage increases, the number and size of the separated spherical particles decrease (Figure 15(a_1_–a_3_)). At the same time, the melting degree of the coating surface becomes more apparent, and the surface roughness gradually decreases. Chen et al. found a similar trend in the research of Ni coatings prepared at different voltages [85]. When the deposition voltage is 110 V, the coating surface presents a particle morphology with an obvious boundary (Figure 15(b_1_)). When the voltage rises to 125 V, the melting patterns on the coating become highly noticeable, and the coating becomes dense (Figure 15(b_2_)). Further voltage escalation leads to micropores forming due to plasma discharge (Figure 15(b_3_)). Hence, an operational voltage higher than the critical voltage of approximately 10–20 V is commonly employed to achieve a smooth and compact metal coating.

In addition to the constant voltage deposition strategy, Smith et al. conducted a study on the deposition of Ni coatings using a stepped voltage [92]. Two different voltage strategies were used to deposit a Ni coating on aluminum substrates, one at constant voltage deposition at 185 V and the other with stepped voltage deposition at 210 V/185 V. The results showed that deposition at a constant voltage of 185 V formed a dense, continuous surface coating consisting of nearly 100% Ni. When using stepped voltage deposition, the high voltage deposition stage at 210 V pre-formed a metal intermetallic compound coating composed of Ni and Al on the coating surface. Due to the addition of an intermediate layer, the stepped voltage deposition coating has a denser appearance, more uniform thickness, and fewer microcracks.

(2) Duty cycle and frequency

Duty cycle and frequency are essential parameters for pulse CPET. In general, using pulse power supply mode can make the coating more uniform and have ideal performance. At present, the waveform of pulsed CPET is mainly a square wave. A square-wave pulse power supply typically includes several critical pulse technical parameters, such as pulse on-time (*t_on_*), pulse interval (*t_off_*), pulse period (*T*), pulse frequency (*f*), and pulse duty cycle (*D*). Equations (1)–(4) illustrate the general method for calculating the parameters of a square-wave power supply [93].

(1) $$\;D=\frac{t_{on}}{T}$$

(2) $$f=\frac{1}{T}$$

(3) $$T=t_{on}+t_{off}$$

(4) $$i_{a}=i_{p}D$$

**Figure 16:**
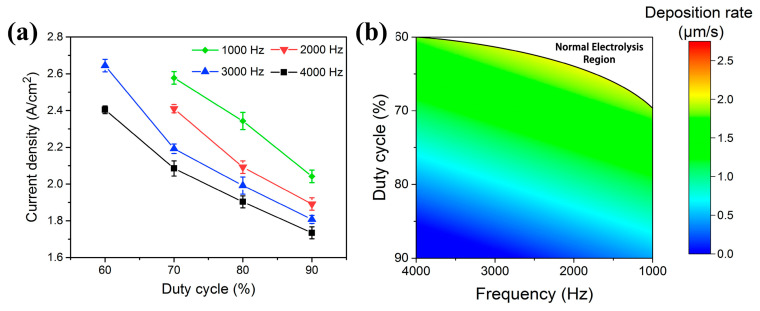
Effect of duty cycle and frequency on (**a**) average current density and (**b**) deposition rate [82].

Yang et al. extensively discussed the influence of duty cycle and frequency on Zn coating deposition using CPET [82]. By adjusting the pulse parameters, the morphology and quality of the Zn coating can be effectively controlled. According to Figure 16a, the average current density decreases as the duty cycle and frequency increase, indicating that lower frequencies and duty cycles result in more pronounced plasma discharge and deposition processes. Yang et al. suggest that adjusting pulse parameters changes the duration of a single pulse, thereby impacting the deposition process of each coating sublayer. Lower duty cycles or frequencies promote thicker coatings but lead to looser and more porous coatings. Higher pulse duty cycles and frequencies lead to denser but thinner coatings. Figure 16b illustrates the connection between deposition rate and frequency (duty cycle) through linear regression analysis. The plasma discharge becomes unstable for sufficiently low frequency or duty cycle (*D* < 0.6, F = 4 kHz or *D* < 0.68, F = 1 kHz), resulting in normal electrolysis.

Zhao et al. utilized high-frequency pulse power to fabricate Ni coatings via CPET [94]. The findings indicate that as the duty cycle increases, there is a decrease in the molten degree of the surface coating, while both the hardness and adhesion of the coating exhibit an increasing trend. With the rising pulse frequency elevation, the coating surface’s molten degree gradually diminishes, leading to a denser and smoother coating structure. Zhao et al. postulated that duty cycle and frequency alterations impact the peak current density in the deposition process. A rise in duty cycle and frequency will reduce peak current density, consequently lowering the melting degree of the coating. Furthermore, the investigations conducted by Zhuang [91] and Yao [95] et al. propose that by employing an appropriate combination of duty cycle and frequency, the surface roughness of metal coatings produced via CPET can be regulated.

Although researchers have different understandings of how duty cycle and frequency affect the deposition process of metal coatings, the effects of duty cycle and frequency show a relatively consistent impact in different metal coating preparation processes. Table 2 summarizes the general rules of changes in porosity, roughness, thickness, adhesion, and melt degree of metal coatings with increased voltage, duty cycle, and frequency.

### 6.3. Other Parameters

(1) Area ratio of anode and cathode

Since the discovery of the cathode plasma discharge phenomenon, how to ensure that the discharge occurs stably at the cathode instead of the anode has become the focus of research. According to Hickling and Sengupta’s study, the location of discharge is mainly determined by the conductivity of the region near the cathode and anode, and the side with lower conductivity tends to be the location of the discharge [30,96]. Zhao et al. studied the relationship between discharge location and the area ratio of cathode and anode in a 0.5 mol/L NaCl solution [42]. The results show that alternating cathode and anode discharge occurs when the cathode-to-anode area ratio is 1.7:1. When the ratio is less than 1.7, only cathode discharge occurs. When the ratio is greater than 1.7, only anode discharge occurs. Changing the cathode’s and anode’s absolute areas has no significant effect on the discharge location under a constant relative area ratio. Zhao et al. suggested that whenever the anode precipitates a unit amount of O_2_, the cathode must precipitate twice as much H_2_. The condition for the alternating plasma discharge of the cathode and anode is that the rate of gas precipitation per unit area of the cathode and anode is equal. Under ideal conditions, the critical area ratio of the cathode and anode is 2. However, due to the slight chlorine evolution reaction on the anode during electrolysis in a NaCl solution and considering the influence of a small amount of water vapor in the gas film, the cathode-to-anode area ratio must be less than 2 when the gas deposition rates are equal. The measured value of 1.7 confirms the correctness of the analysis above.

The “large anode and small cathode” strategy is often adopted in practical production and experiments. On the one hand, it ensures the stable occurrence of cathode plasma discharge. On the other hand, when the cathode area is much smaller than the anode area, the electric field lines will be more concentrated near the cathode surface, resulting in a higher electric field strength on the cathode surface, promoting the growth of deposits on the cathode surface.

(2) Electrode material and surface roughness

The crystal structure and properties of the coating are closely related to the surface roughness of the substrate before deposition. For example, during the plasma electrolytic oxidation of an aluminum alloy, the pre-fabricated texture stimulates increased micro discharge, facilitating the interaction between the coating and the substrate interface, thereby enhancing the mechanical properties of the coating. Similarly, the surface roughness of the substrate significantly impacts the structure and properties of the metal coating produced by CPET.

Gupta et al. deposited a Mo coating on a 4330 V steel substrate [23]. Before deposition, the substrate surfaces with different roughness were prepared by mechanical polishing and plasma cleaning. Obviously, the surface of the substrate after plasma cleaning pre-treatment will induce a dense micro-crater texture, which significantly increases the roughness of the substrate surface. The results show that the final surface roughness of the Mo coating depends on the initial surface roughness of the substrate. The obtained surface profile can be described as the superposition of the roughness caused by the CPET process on the substrate material profile. The hardness test shows that the Mo coating deposited after plasma cleaning pre-treatment has higher hardness.

**Figure 17:**
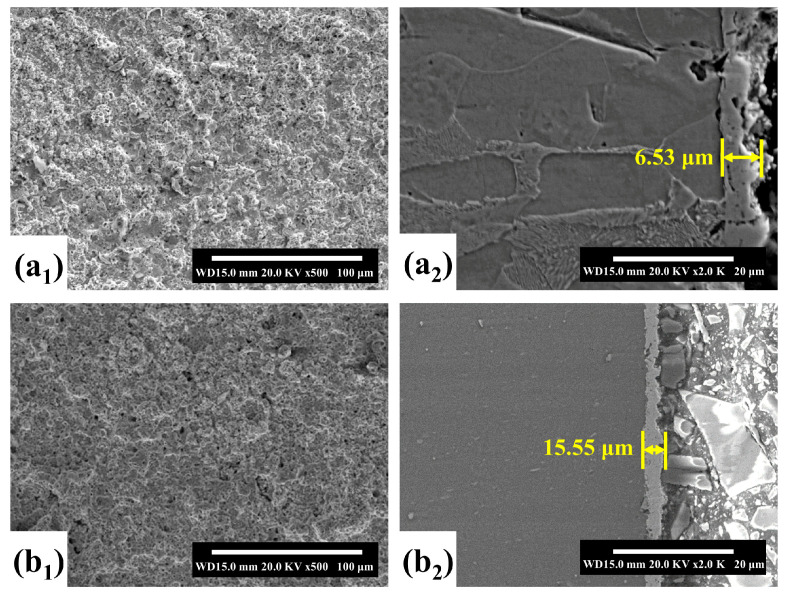
Surface topography and cross-sectional morphology of Ni coatings prepared on different substrates: (**a_1_**,**a_2_**) 1018 steel; (**b_1_**,**b_2_**) 1100 aluminum [92].

Besides the substrate surface roughness, the difference in physical characteristics between the coating and the substrate material also significantly influences the quality of the coating. Smith applied Ni coatings on two different substrates (1018 steel and 1100 aluminum) [92]. Both substrates underwent the same pre-treatment procedure and used the same electrolyte (20 wt.% NiSO_4_) for deposition above the critical voltage. Figure 17 illustrates the surface topography and cross-sectional morphology of Ni coatings applied on distinct substrates. Ni coatings on 1100 aluminum substrates exhibit superior quality regarding surface uniformity, roughness, and coating thickness (Figure 17(b_2_)). Cristian suggests that the difference in melting temperatures (T_m_) between the coating material and the matrix material influences the coating topography and cross-section quality. When the T_m_ of the two materials is similar (e.g., Fe and Ni), the high deposition temperature results in the complete fusion of the materials, while the plasma discharge-induced shock wave leads to increased porosity and an uneven coating [28].

(3) Deposition time

**Figure 18:**
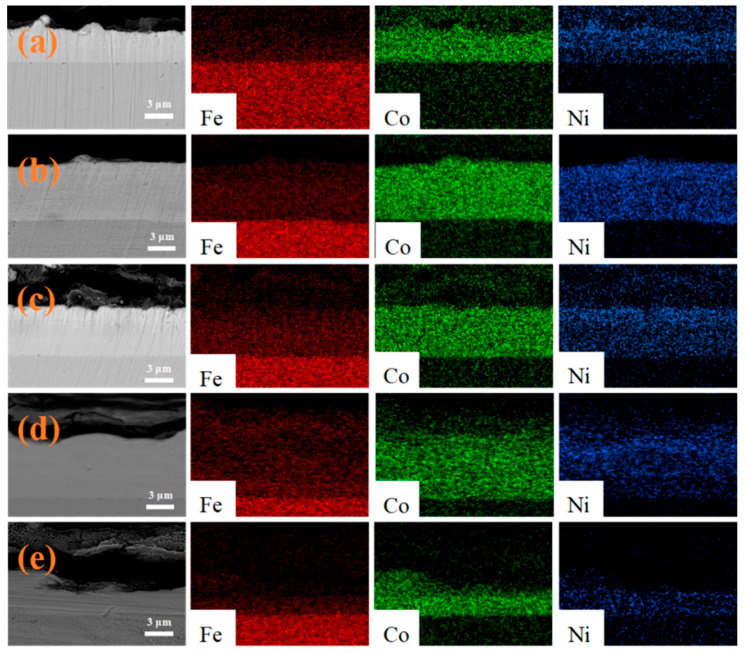
Effect of deposition time on the cross-section of an FeCoNi coating:(**a**) 1 min; (**b**) 2 min; (**c**) 3 min; (**d**) 4 min; (**e**) 5 min [91].

A straightforward way to increase the thickness of the metal coating prepared by CPET is to extend the deposition time. According to the research on FeCoNi coating preparation by Yao et al. [95], when the deposition time was 10 s, the coating surface was relatively smooth, and the coating thickness was about 8 μm. By extending the deposition time to 3 min, the coating thickness reached about 16 μm, and the surface of the sample was further roughened. Although the extended time can effectively increase the thickness of the coating, the thickness of the coating does not continue to grow or even decrease after deposition for more than 5 min. As shown in Figure 18, the study of Zhuang et al. showed similar results [91]. In addition, with the extension of the deposition time, the surface melting degree of the coating gradually increases, and the coating develops holes, cracks, and even delamination.

Currently, the research on long-term CPET deposition (>10 min) remains relatively unexplored, lacking a viable model to elucidate the reason for the limited value of coating thickness. According to the changing trend of surface roughness with varying deposition times, it is evident that prolonged deposition leads to increased roughness, along with a rise in micropores and dendritic protrusions from plasma discharge. The increase in surface roughness inevitably leads to a rise in the actual surface area of the coating surface, thereby affecting the gas evolution and plasma discharge processes during the CPET process. Moreover, heightened surface roughness amplifies tip discharge effects [97], concentrating plasma discharge in defect regions like micropores and dendritic protrusions. Consequently, variations in specific surface area and tip discharge effects likely play pivotal roles in the challenges of thickening coatings.

## 7. Plasma Interaction with Materials Surface

**Figure 19:**
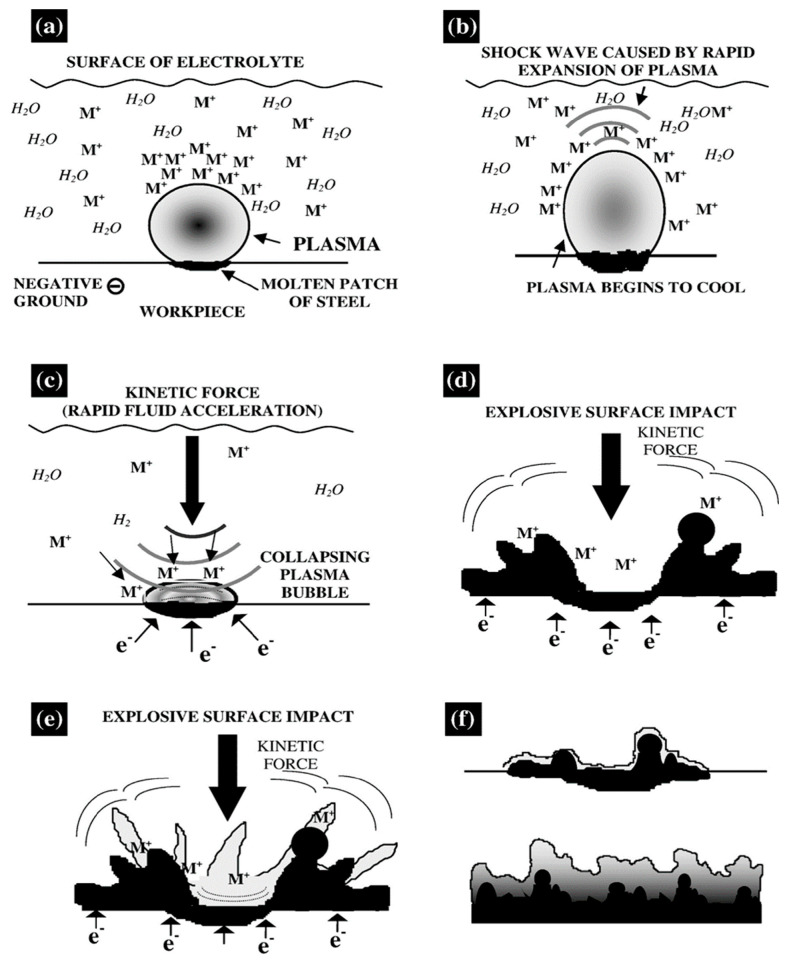
Schematic diagram of CPET deposition mechanism [9]: (**a**) The formation of plasma; (**b**) Shockwave production by the cooling plasma bubble; (**c**) Collapse of plasma bubbles; (**d**) Rupture of plasma bubbles; (**e**) Formation of metal coatings; (**f**) Thickening of metal coating.

The single-bubble breakdown theory is the widely accepted deposition mechanism for preparing metal coatings by CPET [9]. Figure 19 illustrates the interaction between plasma and material surface based on the single-bubble breakdown theory. The defects, such as pits and cavities on the cathode, provide nucleation sites for the formation of gas bubbles captured. As the amount of gas captured near the nucleation site increases, a separate bubble gradually forms. After the bubble is formed, the metal ions move around the bubble and attach to the surface of the bubble under the action of the electric field force. Due to the isolation of bubbles, the electric field strength between the electrolyte and the cathode is high, up to 10^5^ V/m or more. When the critical voltage for bubble breakdown is reached, the gas inside the bubble is ionized, forming a plasma and starting to discharge (Figure 19a). When the bubble is broken down by discharge, the metal ions attached to the bubble surface are accelerated to the cathode surface (Figure 19b–d)). At the same time, the kinetic energy generated by rupture is released to the surface of the cathode in the form of a shock wave (energy can reach hundreds of megapascals). The combined effect of plasma acceleration and shock wave enhancement leads to a relatively high deposition rate of metal ions. After bubble rupture, the electrode surface is quenched by the electrolyte environment, forming a unique surface microstructure (Figure 19e,f). Due to periodic surface melting and quenching cooling, the coating has good adhesion and ultrafine grain structure. The above deposition mechanism based on single bubbles reasonably explains the morphological characteristics of metal coatings prepared by CPET. Still, plasma discharge during the actual deposition process is more complicated. Belkin argues that the theoretical mechanism of this single bubble is suitable for explaining discrete plasma discharge, but it cannot reasonably describe the discharge process in the presence of gas films [11].

**Figure 20:**
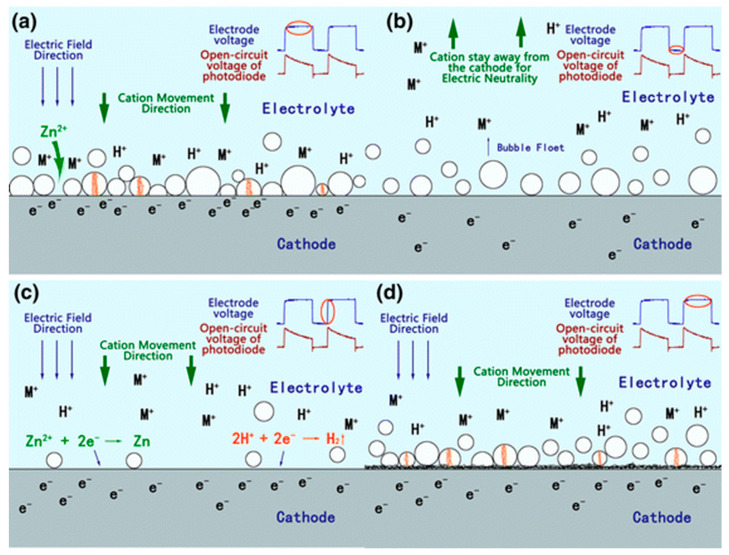
Coating deposition process in a single pulse stage: (**a**) High-level stage of the previous pulse; (**b**) Low-level stage of the previous pulse; (**c**) Rising stage of the next pulse; (**d**) High-level stage of the next pulse [87].

Yang et al. explored the deposition mechanism of Zn coatings in pulsed mode based on the single bubble theory in conjunction with the photovoltage of the plasma process [87]. As shown in Figure 20a, the breakdown of bubbles by plasma mainly occurs in the high-voltage stage of a single pulse, and no apparent deposits were found at this stage. Between pulses, there is no photovoltage present between the electrodes. The metal ions accumulated on the cathode surface move away from the cathode surface to maintain the solution’s electroneutrality, as shown in Figure 20b. For bubbles, the previous bubbles gradually move away from the surface at this stage. Upon the next pulse, a complete gas film structure must be re-established on the cathode surface, requiring time from voltage application to plasma generation. During this time, rapid electrolysis occurs on the cathode surface to obtain a large number of bubbles, thus blocking the pathway of ions, and the deposition of Zn occurs mainly at this stage (Figure 20c,d). Yang et al. clarified that plasma does not enhance metal ion deposition but serves to clean the cathode surface. The deposition of metallic Zn on the cathode surface mainly originated from the conventional cation electrolytic reduction reaction, contrary to Paulmier’s perspective. Paulmier investigated the relationship between plasma discharge and coating formation and showed that no deposits would be observed without the presence of plasma discharge [98].

At present, most of the research on CPET is from the perspective of application, and there is no systematic study on the properties and mechanism of plasma. Due to the existence of gas films, the physical and chemical processes of CPET are relatively complex, and no theoretical model has been formed that can profoundly reveal the plasma formation and interaction with the materials. From this point of view, it is urgent to strengthen the research on the characteristics and mechanism of CPET.

## 8. Conclusions and Outlook

The CPET process is a promising surface modification and coating preparation technology. Due to the synergistic effect of periodic melting, quenching, and cooling of the substrate surface, the CPET process can quickly and effectively achieve the metal’s cleaning, modification, and coating. Through the investigation of previous literature, this paper briefly summarizes the principle, application, and development status of the CPET process. In addition, the effects of electrolyte parameters, electrical parameters, and process parameters on plasma discharge and coating deposition were summarized in detail in the field of the CPET process for preparing metal coatings. As far as the current focus of scholars is concerned, there is still room for further scope in this field, with the following objectives.

(1) Detailed reports have been made on the effects of process parameters and electrolytes on the CPET process, particularly on coating morphology and properties. However, there are different theoretical views on the CPET deposition process, and a model revealing plasma formation and substrate interaction mechanisms is lacking. Thus, further research on liquid-phase discharge plasma characteristics and generation mechanisms is imperative.
(2) Current research focuses on the single influencing factors of CPET, with few studies investigating the interactions between these factors. In addition, due to the complexity of discharge in liquids, there is a lack of direct observation and characterization of plasma in CPET. Therefore, combining the evolution of current and voltage during the discharge process and observation methods such as photovoltage and spectroscopy is beneficial for better understanding and establishing the relationship between plasma discharge and CPET processes.
(3) Various factors influence the quality and performance of CPET coatings. All parameters affecting the deposition process point to a core factor: the stability and thickness of the gas film during CPET are crucial for controlling coating quality. Currently, research on constraining the gas film in CPET coating preparation is lacking, with no systematic study attempting direct control of the gas film to enhance process repeatability and stability. Thus, in-depth research on gas film characteristics and methods for constraining it is necessary to achieve controllable CPET processing.
(4) Although the CPET process has many advantages, its application in large-scale industrial manufacturing still faces difficulties. The main reason is that the CPET process involves the establishment and breakdown of the gas film, resulting in high energy consumption during the deposition process. Therefore, it is crucial to reduce the energy consumption of CPET through device design and process optimization.
(5) The stability and final outcome of the CPET process are affected by various factors. Optimizing one parameter can lead to chain changes in other parameters. For instance, altering the electrolyte’s pH or the concentration of metal salts can change the voltage required for plasma formation. Consider combining machine learning with CPET parameter optimization to achieve objective optimization under multi-parameter coupling.
(6) In large-scale industrial manufacturing, it is considered to deeply integrate CPET equipment with precision numerical control systems, intelligent robots, and advanced sensors to build an advanced and intelligent CPET system.

## Figures and Tables

**Table 1:** Electrolyte and process parameters for the preparation of various metal coatings.

Coating	Substrate	Voltage	Electrolyte	Researchers
Ni	Aluminium alloy	100 V/80%/1000 Hz	30 g/L NiSO_4_ + 0.4 mol/L H_2_SO_4_	Zhang [22]
	Copper sheet	-/-/-	100 g/L NiSO_4_ + 40 g/L H_2_SO_4_	Zhao [83]
	1018 steel	DC 200 V	20 wt.% NiSO_4_	Smith [86]
	Steel	DC 200 V	20 wt.% NiSO_4_·6H_2_O	Cionea [28]
Zn	Stainless steel	DC 180 V	21 wt.% ZnSO_4_	Meletis [34]
	Q195 wire	120 V/80%/4000 Hz	21 wt.% ZnSO_4_	Yang [87]
	Steel	DC 170 V	16 wt.% ZnSO_4_·7H_2_O	Cionea [28]
Ag	316 stainless steel	200/25%/50 kHz	0.03 wt.% AgNO_3_ + 20 mL NH_3_·H_2_O	Lin [84]
Co	304 stainless steel	DC 85 V	25 g/L CoSO_4_ + 60 g/L H_2_SO_4_	Quan [88]
Mo	Steel	DC 170 V	10 wt.% Na_2_MoO_4_	Cionea [28]
Ni-20%Cr	304 stainless steel	DC 80–100 V	20 g/L NiSO_4_ + 20 g/L Cr_2_(SO_4_)_3_ +40 g/L H_2_SO_4_	Quan [76]
FeCoNi	304 stainless steel	150 V/70%/1000 Hz	10 g/L FeSO_4_ + 10 g/L CoSO_4_+ 10 g/L NiSO_4_ + 10 mL/L H_2_SO_4_	Xia [89]

**Table 2:** The influence of electrical parameters on the quality of the coating.

Parameters	Porosity	Roughness	Thickness	Bond Strength	Melting Degree
Voltage ↑	↑	↓	↓	↑	↑
Duty cycle ↑	↓	↓	↓	↑	↓
Frequency ↑	↓	↓	↓	↑	↓

↑ and ↓ represent increase and decrease, respectively.

## Data Availability

No new data were created or analyzed in this study.
